# 

**Published:** 1908-09-19

**Authors:** 


### CONTENTS.

| | PAGE |
|---|---|
| **The British Association and Anæsthetics** | 643 |
| **The Lunacy Commission** | 644 |
| **Annotations—** | |
| Educational Science—Newspaper Medicine—The American Hospital Conference | 645 |
| **Medical Opinion and Movement** | 646 |
| **Hospital Clinics—** | |
| The Injuries Incidental to Athletic Exercises. By R. H. A. Whitelocke, M.D. (Edin.), F.R.C.S. | 649 |
| Parotitis after Operations | 652 |
| **Medicine—** | |
| Pigmentation and the Diagnosis of Addison's Disease | 653 |
| Enlargements of the Spleen—VII. | 654 |
| **Surgery—** | |
| Some Aspects of Intestinal Obstruction | 655 |
| **Obstetrics—** | |
| Points in the Ætiology of Puerperal Sepsis | 656 |
| **Laryngology and Rhinology—** | |
| Enlargement of the Tonsils—II. | 657 |
| **Therapeutics—** | |
| British Pharmaceutical Conference | 658 |
| **"The Hospital" Medical Book Supplement.—No. X.** | 659 |
| **Hospital Administration—** | |
| Graduate Study on the Continent—Germany | 663 |
| **Nursing Administration—** | |
| The Domestic Staff—IV. | 665 |
| **News and Coming Events** | 666 |
| **Editor's Letter-Box** | 667 |

